# Supplementary material for: Content-rich biological network constructed by mining PubMed abstracts
Source: BMC Bioinformatics. 2004 Oct 8;5:147. doi: 10.1186/1471-2105-5-147 (PMC528731; doi:10.1186/1471-2105-5-147)
Supplement: Additional File 2 — The original results of the above study (non-essential files are deleted to keep the file size under the limit set by BMC bioinformatics). [file 1471-2105-5-147-S2.bz2 › chilibotAdditionalFile2/dip20/323ID9824158E3491/html/E3L_PRKR.html]

 


 **E3L** and **PRKR** 
  
Found 25 abstracts in PubMed, retrieved 20.  
 

 What does Google say? 
 PDF only 
| .edu only 

---

**Interactive relationship** (e.g. stimulation, inhibition, etc)

**Inhibitory relationship**- The VV  **E3L**  protein is a potent inhibitor of PKR  [ **PRKR** ] .  Ref: 11333890 J Virol, 2001
- Vaccinia virus  **E3L**  encodes a dsRNA binding protein that inhibits PKR  [ **PRKR** ]  in virus infected cells, presumably by sequestering dsRNA activators.  Ref: 9819417 Mol Cell Biol, 1998
- The  **E3L**  gene products, p25 and p20, act as inhibitors of PKR  [ **PRKR** ] , presumably by binding and sequestering activator dsRNA from the kinase.  Ref: 7527085 J Virol, 1995
- These results suggest that a novel cellular protein that is activated by viral products in addition to dsRNA and is sensitive to  **E3L**  inhibition is responsible for IRF activation and reveal a novel mechanism for the anti IFN effect of  **E3L**  distinct from its inhibition of PKR  [ **PRKR** ] .  Ref: 11124948 J Biol Chem, 2001
- **E3L**  and K3L markedly reduced the activation of the double stranded RNA dependent protein kinase, PKR  [ **PRKR** ] , and led to a significant reduction in apoptosis in HeLa cells.  Ref: 11878896 Virology, 2001
- The vaccinia virus VV  **E3L**  gene encodes a dsRNA binding protein that inhibits activation of the IFN induced, dsRNA dependent protein kinase, PKR  [ **PRKR** ] , the 2 5A synthetases RNase L system and other dsRNA dependent pathways, thus leading to efficient VV replication.  Ref: 12466958 Oncogene, 2002
- This assay is based on the use of cells derived from homozygous PKR  [ **PRKR** ]  knockout mice Pkr  [ **PRKR** ]  infected with a VV mutant lacking  **E3L**  delta  **E3L**  and transiently transfected with a luciferase reporter gene together with plasmid vectors expressing 2 5A synthetase, RNase L, or  **E3L** , all controlled by the same inducible promoter.  Ref: 9568039 Virology, 1998
- However, unlike most poxviruses, MCV lacks a homologue to the  **E3L**  and K3L proteins of vaccinia virus, which are involved in the control of the key antiviral and pro apoptotic dsRNA dependent protein kinase, PKR  [ **PRKR** ] .  Ref: 11714980 J Gen Virol, 2001
**Neutral relationship**- To explore how  **E3L**  enables vaccinia virus to evade the interferon system, cells and mice deficient in the principal interferon regulated antiviral enzymes, PKR  [ **PRKR** ]  and RNase L, were infected with wild type vaccinia virus and strains of vaccinia virus from which  **E3L**  had been deleted  **E3L**  deleted strains.  Ref: 11967338 J Virol, 2002
- IFN pretreated cells infected with VV in which the  **E3L**  locus was replaced with the NS5A gene VVNS5A displayed diminished phosphorylation of PKR  [ **PRKR** ]  and eIF2alpha in a transient manner.  Ref: 11333890 J Virol, 2001
- The vaccinia virus  **E3L**  gene product interacts with both the regulatory and the substrate binding regions of PKR  [ **PRKR** ]  implications for PKR  [ **PRKR** ]  autoregulation.  Ref: 9792841 Virology, 1998
- Accordingly, infection of IFN pretreated HeLa S3 cells with an  **E3L**  deficient VV VVDeltaE3L resulted in increased phosphorylation levels of both PKR  [ **PRKR** ]  and eIF2alpha.  Ref: 11333890 J Virol, 2001

**Non-interactive relationship** (e.g. studied together, co-existance, homology, etc.)

- While wild type virus was unaffected by RNase L and PKR  [ **PRKR** ] , virus lacking  **E3L**  replicated only in the deficient cells.  Ref: 11967338 J Virol, 2002
- Infection of L929 cells with  **E3L**  minus virus led to rRNA degradation typical of activation of the 2 5 oligoadenylate synthetase RNase L system, and extracts of infected cells lacked the PKR  [ **PRKR** ]  inhibitory activity characteristic of wild type VV.  Ref: 7527085 J Virol, 1995
- a deletion mutant of vaccinia virus VP1080 lacking the PKR  [ **PRKR** ]  inhibitory polypeptide  **E3L**  induced the expression of germ line epsilon.  Ref: 9445015 J Virol, 1998
- In contrast, other inhibitors of PKR  [ **PRKR** ]  including vaccinia virus  **E3L**  and K3L gene products, and the HIV trans activating response TAR element binding protein TRBP, did not disrupt ribosome association of PKR  [ **PRKR** ] .  Ref: 9753471 Biochemistry, 1998
- Nonphosphorylatable mutant eIF2alpha, knockout of PKR  [ **PRKR** ]  and PKR  [ **PRKR** ]  inhibitors 2 aminopurine, transdominant negative PKR  [ **PRKR** ] , or vaccinia  **E3L**  correspondingly enhances translation of IFN gamma mRNA.  Ref: 11832212 Cell, 2002
- The vaccinia virus  **E3L**  gene product, pE3, is a dsRNA binding protein that prevents activation of the interferon induced, dsRNA activated protein kinase, PKR  [ **PRKR** ] .  Ref: 9792841 Virology, 1998
- Taken together, the results indicate that the OVIFNR gene functions as an interferon resistance gene, the product of which inhibits PKR  [ **PRKR** ]  in a similar way to the vaccinia virus  **E3L**  gene product.  Ref: 9640243 Immunology, 1998
- Vaccinia virus was able to overcome these cellular variations by regulating PKR  [ **PRKR** ]  activity through the synthesis of either  **E3L**  or K3L.  Ref: 12167348 Virology, 2002
- The vaccinia virus VV  **E3L**  gene, which encodes a potent inhibitor of the interferon IFN induced, double stranded RNA dsRNA dependent protein kinase, PKR  [ **PRKR** ] , is thought to be involved in the IFN resistant phenotype of VV.  Ref: 7527085 J Virol, 1995
- Nevertheless,  **E3L**  deleted virus failed to replicate to high titers or to cause significant morbidity or mortality in triply deficient mice lacking RNase L, PKR  [ **PRKR** ] , and Mx1.  Ref: 11967338 J Virol, 2002
- The role of the PKR  [ **PRKR** ]  inhibitory genes,  **E3L**  and K3L, in determining vaccinia virus host range.  Ref: 12167348 Virology, 2002
- Despite evidence for a lack of involvement of dsRNA and PKR  [ **PRKR** ] , vaccinia virus  **E3L**  protein, a dsRNA binding protein capable of inhibiting PKR  [ **PRKR** ] , was an effective IRF3 and 7 phosphorylation inhibitor.  Ref: 11124948 J Biol Chem, 2001
- Data obtained from infection of epithelial cells with a vaccinia virus lacking the PKR  [ **PRKR** ]  inhibitory polypeptide,  **E3L** , revealed that PKR  [ **PRKR** ]  activation was essential for TNF alpha but not for IL 1 beta expression.  Ref: 12055262 J Immunol, 2002
- The vaccinia virus  **E3L**  gene codes for double stranded RNA dsRNA binding proteins which can prevent activation of the dsRNA dependent, interferon induced protein kinase PKR  [ **PRKR** ] .  Ref: 9032331 J Virol, 1997
